# CHIP ameliorates cerebral ischemia-reperfusion injury by attenuating necroptosis and inflammation

**DOI:** 10.18632/aging.203774

**Published:** 2021-12-14

**Authors:** Dabao Yao, Shuo Zhang, Zhengwei Hu, Haiyang Luo, Chengyuan Mao, Yu Fan, Mibo Tang, Fen Liu, Si Shen, Liyuan Fan, Mengjie Li, Jingjing Shi, Jiadi Li, Dongrui Ma, Yuming Xu, Changhe Shi

**Affiliations:** 1 Department of Neurology, The First Affiliated Hospital of Zhengzhou University, Zhengzhou University, Zhengzhou 450000, Henan, China; 2 Henan Key Laboratory of Cerebrovascular Diseases, The First Affiliated Hospital of Zhengzhou University, Zhengzhou University, Zhengzhou 450000, Henan, China; 3 Institute of Neuroscience, Zhengzhou University, Zhengzhou 450000, Henan, China

**Keywords:** cerebral ischemia-reperfusion injury, CHIP, MCAO, necroptosis, inflammation

## Abstract

Blood reperfusion of ischemic cerebral tissue may cause cerebral ischemia-reperfusion (CIR) injury. Necroptosis and inflammation have been demonstrated to be involved in the disease-related process of CIR injury. The E3 ubiquitin ligase carboxyl terminus of Hsp70-interacting protein (CHIP) can modulate multiple cellular signaling processes, including necroptosis and inflammation. Numerous studies have demonstrated the neuroprotective effects of CHIP on multiple central nervous system (CNS) diseases. However, the effects of CHIP on CIR injury have not been fully explored. We hypothesize that CHIP can exert neuroprotective effects by attenuating necroptosis and inflammation during CIR injury. In the present study, adult wild-type (WT) C57BL/6 mice and CHIP knock-in (KI) mice with a C57BL/6 background and CHIP overexpression in neural tissue underwent middle cerebral artery occlusion (MCAO) surgery to simulate CIR onset. Our data indicated that CHIP expression in the peri-infarct tissue was markedly increased after MCAO surgery. Compared with WT mice, CHIP KI mice significantly improved neurological deficit scores, decreased cerebral infarct volume, and attenuated brain edema and neuronal damage. Meanwhile, CHIP overexpression attenuated necroptosis and inflammation induced by MCAO surgery. These findings indicated that overexpression of CHIP might exert neuroprotective effects by attenuating necroptosis and inflammation during CIR injury, and increasing CHIP levels may be a potential strategy in cerebrovascular disease therapy.

## INTRODUCTION

Stroke is a serious cerebrovascular disease associated with aging, and approximately 80% of stroke events are classified as ischemic strokes [1]. The recovery of neurological function after ischemic stroke onset requires restoring the blood supply of the ischemic brain tissue as early as possible. Mechanical thrombectomy and thrombolytic therapy using tissue plasminogen activator are two common methods to restore blood supply of ischemic brain tissue in ischemic stroke treatments [2]. However, achieving blood reperfusion of the ischemic brain tissue by mechanical thrombectomy or thrombolytic therapy may cause secondary brain injury, also known as cerebral ischemia-reperfusion (CIR) injury [3]. Studies have shown that CIR injury involves multiple mechanisms, such as mitochondrial damage, energy failure, calcium overload, and inflammation [4–6]. These mechanisms can induce neuronal cell death, which causes neurological deficits. Thus, strategies to reduce neuronal cell death may be conducive to treating brain injury after CIR.

Necroptosis is a programmed and regulated cell death and an important pathological foundation of many diseases, such as neurodegenerative diseases, ischemia-reperfusion injury, and inflammation [7–9]. Necroptosis is mainly regulated by receptor-interacting protein kinase 1 (RIPK1), receptor-interacting protein kinase 3 (RIPK3), and mixed lineage kinase domain-like pseudokinase (MLKL) [10]. Certain stimulating factors, such as ischemia-reperfusion, can initiate the tumor necrosis factor-α (TNF-α)-mediated necroptosis signaling pathway, leading to the activation of RIPK1. Activated RIPK1 interacts with RIPK3 and forms necrosomes. Activated RIPK3 in the necrosome phosphorylates MLKL and leads to the formation of oligomers, which translocate to the cytomembrane and cause cytomembrane damage and necroptotic cell death [11]. Several studies have indicated that inhibition of necroptosis can realize neuroprotective effects after CIR in mice, such as reducing cerebral infarct volume and improving motor and cognitive function [12, 13]. Therefore, suppression of necroptosis may be an essential method for the treatment of CIR injury.

Studies have demonstrated that inflammation is a significant contributor to CIR injury [14, 15]. A variety of cells, such as microglia and astrocytes, are activated following ischemic stroke [16]. These activated cells release large amounts of inflammatory mediators such as TNF-α, interleukin-1β (IL-1β), and interleukin-6 (IL-6), aggravating brain injury after CIR [16]. Furthermore, necroptosis is considered a type of cell death that can trigger inflammation. The broken necroptotic cells release various cellular contents into the extracellular environment. Some cellular components, such as damage-associated molecular patterns (DAMPs) can activate immune cells and then elicit inflammation [17], which aggravates brain injury following CIR. Multiple studies have also shown that inhibition of inflammation mitigates brain injury after CIR [18–20].

The carboxyl terminus of Hsp70-interacting protein (CHIP) encoded by the *STUB1* gene is an E3 ubiquitin ligase with molecular chaperone and ubiquitin ligase (E3) activity [21, 22]. CHIP participates in cellular protein quality control by regulating the degradation of chaperone-bound proteins [23]. Studies have indicated that CHIP can negatively regulate necroptosis by enhancing the degradation of RIPK1 and RIPK3 [24, 25]. In an *in vitro* cell model, downregulation of CHIP expression by gene knockdown increased the levels of RIPK1 and RIPK3 [24]. CHIP deficient mice exhibit intestinal inflammation and premature death, whereas RIPK3 knockout can reverse these phenotypes [24]. Our previous study demonstrated that CHIP overexpression exerts protective effects by suppressing necroptosis in an oxygen-glucose deprivation (OGD) cell model [25]. Some studies have also shown that CHIP plays an important role in regulating the immune response [26, 27]. CHIP overexpression attenuates myocardial inflammation in a myocardial dysfunction mouse model [28]. These findings suggest the essential role of CHIP in regulating necroptosis and inflammation.

Necroptosis and inflammation are essential pathogenic mechanisms of CIR injury, and CHIP overexpression can attenuate both. Thus, we hypothesize that CHIP overexpression can ameliorate CIR injury by attenuating necroptosis and inflammation. Using a middle cerebral artery occlusion (MCAO) mouse model, the present study examined CHIP expression after CIR. Moreover, CHIP knock-in (KI) mice overexpressing CHIP in neural tissue and age-matched wild-type (WT) mice were subjected to MCAO surgery to explore whether increasing CHIP levels can exert neuroprotective effects and possible mechanisms.

## RESULTS

### CHIP expression was upregulated after MCAO

**Figure 1:**
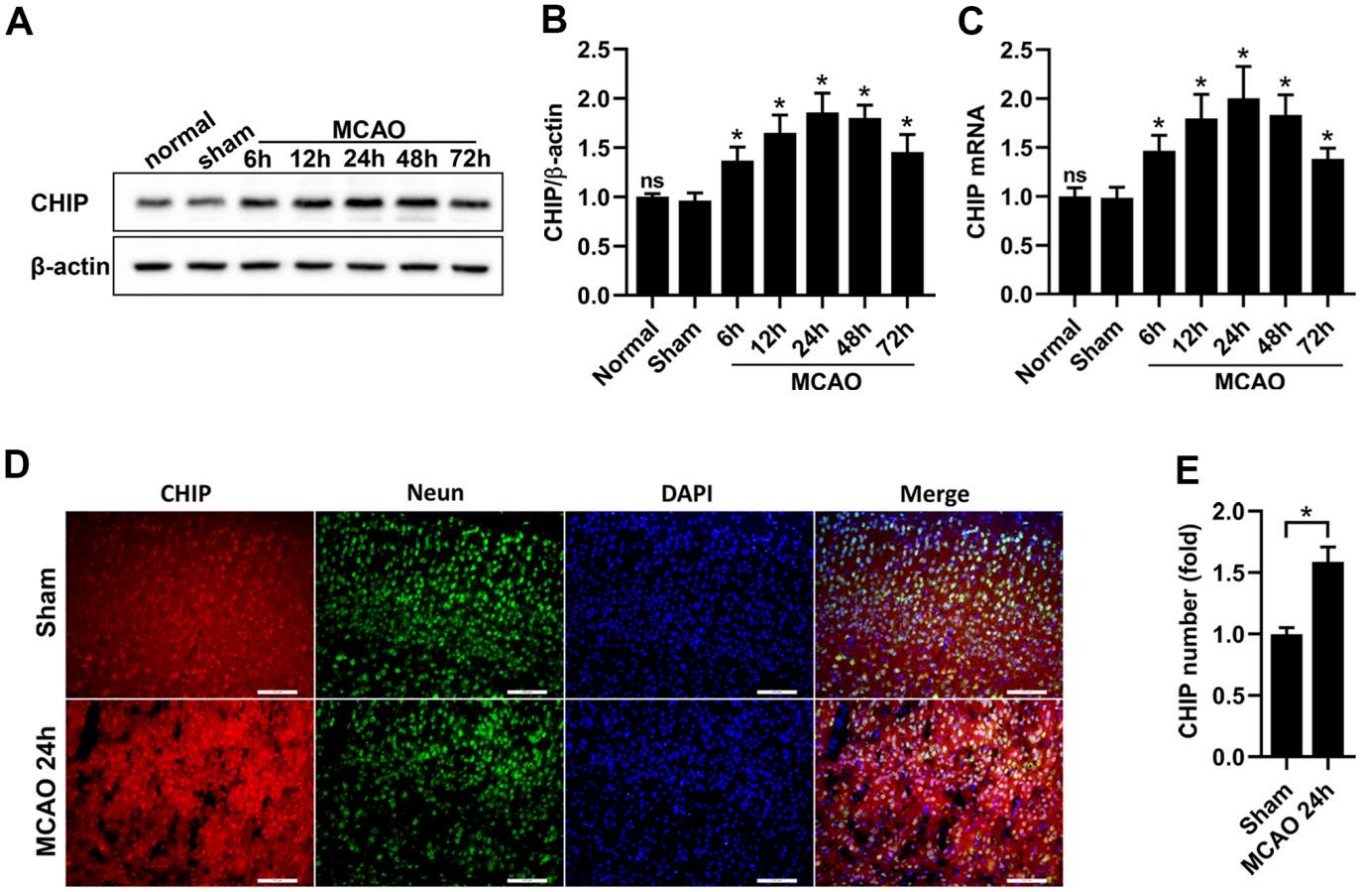
**CHIP levels were increased after MCAO.** (**A**, **B**) Western blot and quantitative analysis of CHIP at different time points after reperfusion in the sham and MCAO surgery groups. (**C**) The mRNA expression of CHIP. (**D**, **E**) Immunostaining and quantitative analysis of CHIP at 24 h after reperfusion in the sham and MCAO surgery groups. Scale bar =100 μm. Data are presented as the mean ± SEM; n=6/group; ns, no significant difference vs. sham group; ^*^P < 0.05 vs. sham group.

CHIP overexpression can exert neuroprotective effects on many central nervous system (CNS) diseases. Studies have indicated that CHIP expression is increased in some chronic neurodegenerative disorders, such as Alzheimer’s disease [29] and spinocerebellar ataxia type 3 [30]. In the present study, we aimed to evaluate the expression of CHIP in acute neurodegenerative disorders. We established MCAO mouse models using WT mice to detect CHIP expression after CIR. Western blotting and reverse transcription-polymerase chain reaction (RT-PCR) results indicated that CHIP levels of the peri-infarct tissues were increased from 6 h to a peak at 24 h and then decreased after MCAO surgery, remaining higher at 72 h after MCAO surgery than sham surgery (Figure 1A–1C). Immunofluorescence staining results indicated that CHIP was colocalized with NeuN (the neuron marker), and CHIP levels were increased in the peri-infarct tissues at 24 h after MCAO surgery compared with sham surgery (Figure 1D, 1E). These results indicated that CHIP expression was upregulated after MCAO, and neurons might be the primary cellular sources for CHIP induction.

### CHIP overexpression in the brain of CHIP KI mice

**Figure 2:**
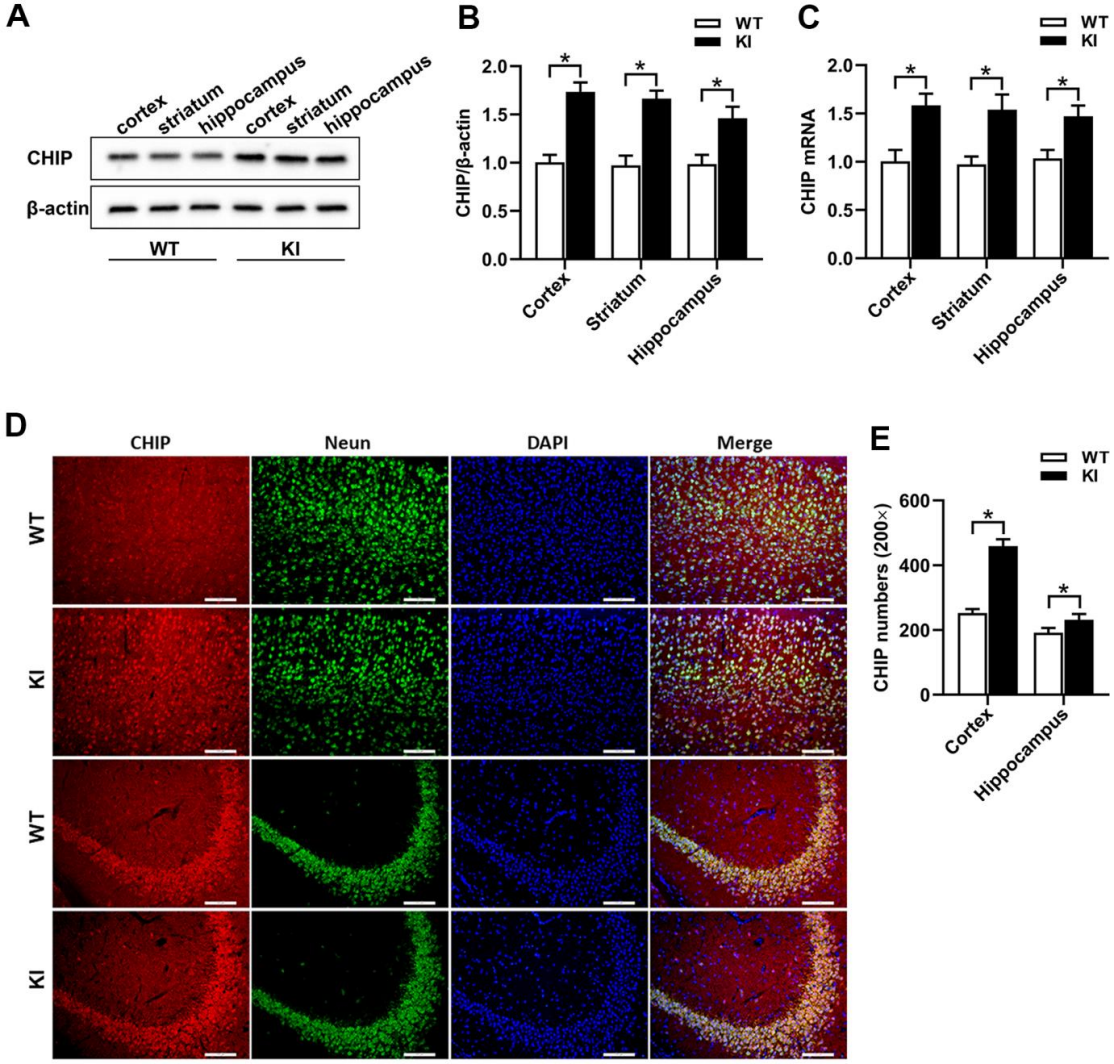
**CHIP overexpression in CHIP KI mice.** (**A**, **B**) Western blot and quantitative analysis of CHIP in the cortex, striatum, and hippocampus. (**C**) The mRNA expression of CHIP. (**D**) Immunostaining of CHIP, NeuN, and DAPI in the cortex and hippocampus of WT mice and CHIP KI mice. (**E**) Quantitative analysis of immunostaining showed the positive numbers of CHIP. WT, WT mice; KI, CHIP KI mice. Scale bar =100 μm. Data are presented as the mean ± SEM; n=6/group; ^*^P < 0.05.

We used CRISPR/Cas9 technology to construct CHIP KI mice carrying an exogenous *STUB1* gene to achieve CHIP overexpression in neural tissue. CHIP KI mice and age-matched WT mice were selected to compare CHIP expression in brain tissue. Western blotting results indicated CHIP overexpression in the cortex, striatum, and hippocampus of CHIP KI mice (Figure 2A, 2B), and CHIP mRNA expression in these tissues showed a similar trend (Figure 2C). Consistently, immunochemical staining results showed that CHIP-positive numbers were significantly higher in the cortex and hippocampus of CHIP KI mice than in WT mice (Figure 2D, 2E). Thus, our results indicated that CHIP levels were markedly higher in CHIP KI mouse brains than in WT mouse brains.

### CHIP overexpression alleviated brain injury after MCAO

**Figure 3:**
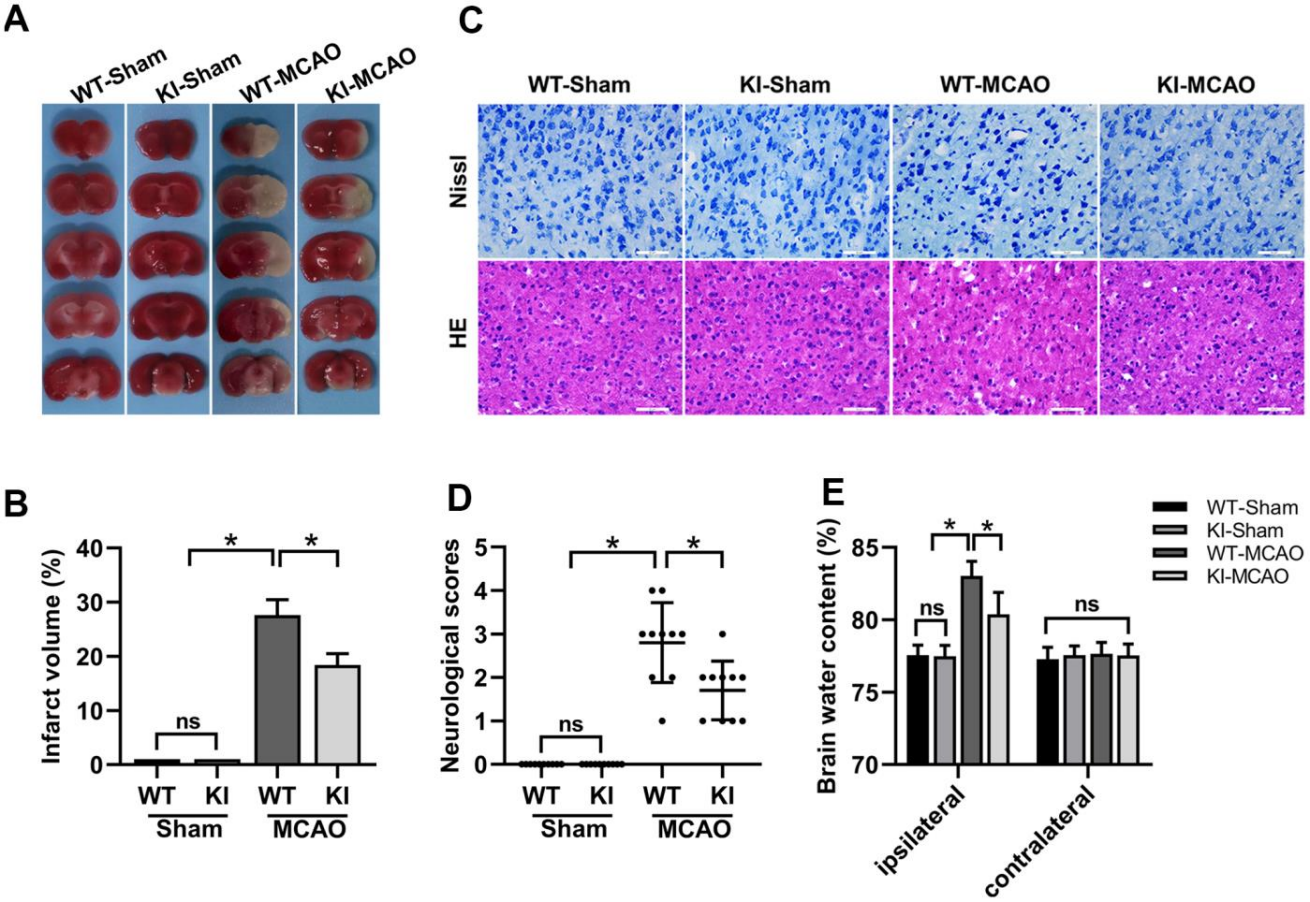
**CHIP overexpression alleviated brain injury after MCAO.** (**A**) TTC staining showed the cerebral infarct volume. (**B**) Quantitative analysis of the cerebral infarct volume. (**C**) HE and Nissl staining indicated the morphological characteristics. (**D**) Neurological scores. (**E**) Brain water content. Scale bar =50 μm. Data are presented as the mean ± SEM; n=6/each group and neurological scores n=10/each group; ns, no significant difference; ^*^P < 0.05.

In the present study, we established MCAO mouse models using WT mice and CHIP KI mice to assess the effect of CHIP overexpression on brain injury after CIR. The cerebral infarct volume, neurological deficit scores, brain edema, and histologic characteristics of the infarct area were detected 24 h after mice were subjected to sham surgery or MCAO surgery. 2,3,5-Triphenyltetrazolium chloride (TTC) staining was performed to measure the cerebral infarct volumes of mice (Figure 3A), and the quantification results showed that the cerebral infarct volumes of CHIP KI mice were significantly decreased compared with those of WT mice after MCAO (Figure 3B). Histologic characteristics of the infarct areas were detected using hematoxylin-eosin (HE) staining and Nissl staining. The HE staining results showed that the cerebral cortex cells of both WT mice and CHIP KI mice were neatly arranged and had normal morphology after sham surgery (Figure 3C), while the cerebral cortex cells of WT mice were loosely arranged, swollen, and nuclear pyknosis after MCAO surgery, and the cerebral cortex cell damage of CHIP KI mice was less after MCAO surgery (Figure 3C). For Nissl staining, a substantial number of neurons in the infarct areas of WT mice were abnormally arranged, narrowed, and deeply stained after MCAO surgery (Figure 3C), while neuronal damage in the cortex of CHIP KI mice was reduced after MCAO surgery (Figure 3C). These results indicated that CHIP overexpression could attenuate cerebral histopathological damage after MCAO surgery. The Longa test was used to assess the neurological deficits of mice, and our results showed that Longa’s neurological scores in CHIP KI mice were lower than those of WT mice after MCAO surgery (Figure 3D), which indicated that overexpression of CHIP could improve the neurological defects in the MCAO mouse model. Brain edema was calculated by brain water content. Our results indicated that the brain water content of the ipsilateral hemisphere increased after MCAO surgery compared with sham surgery, and CHIP overexpression significantly reduced the brain water content of the ipsilateral hemisphere after MCAO (Figure 3E), which indicated that CHIP overexpression could reduce brain edema after MCAO surgery. In addition, the brain water content of the contralateral hemisphere was not significantly different between the MCAO surgery and sham surgery groups (Figure 3E). These results indicated that CHIP overexpression alleviates brain injury after MCAO surgery.

### CHIP overexpression reduced neuronal cell death after MCAO

**Figure 4:**
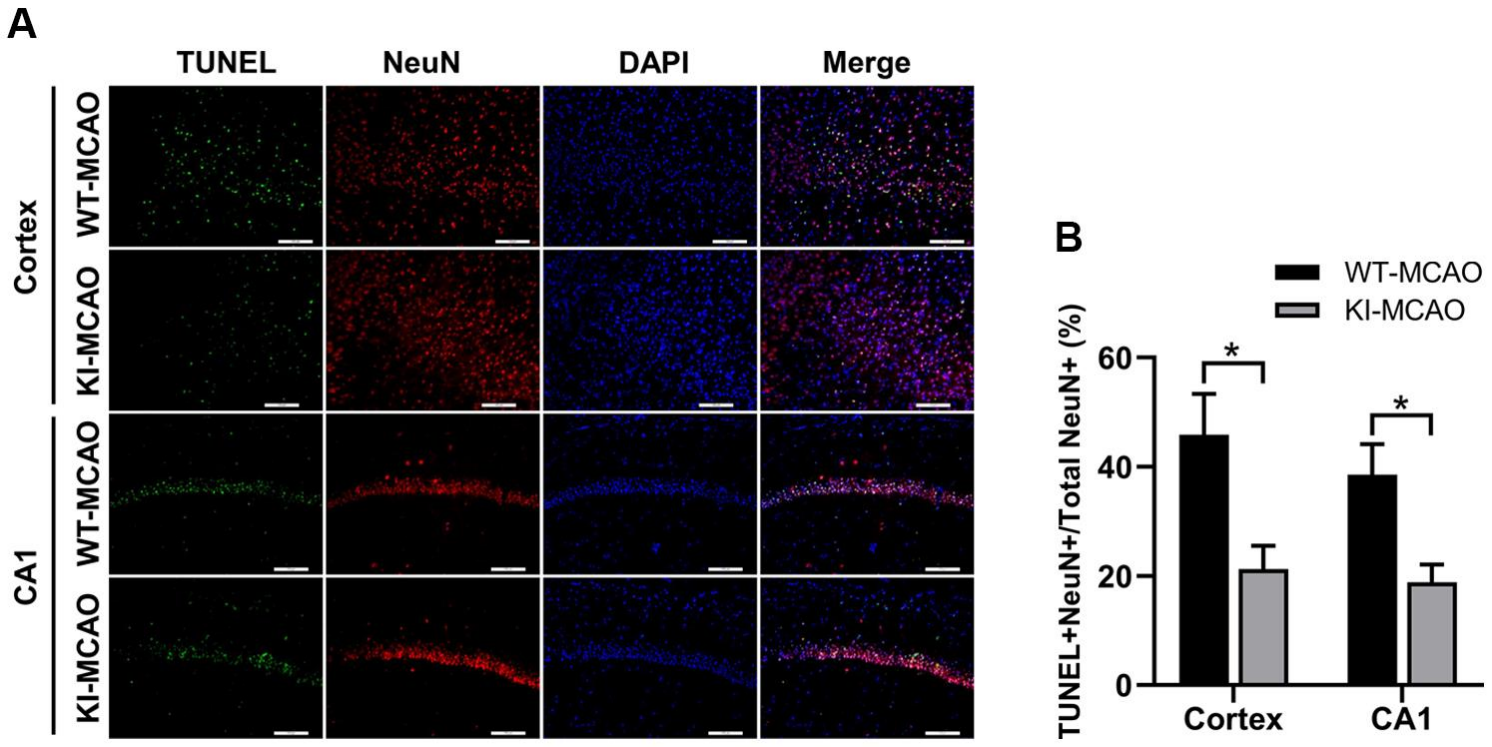
**CHIP overexpression reduced neuronal cell death after MCAO.** (**A**) TUNEL^+^ cells in the cortex and hippocampus CA1 regions were detected by TUNEL/NeuN staining after sham or MCAO surgery. (**B**) Quantitative analysis of total TUNEL^+^ ratio. Scale bar =100 μm. Data are presented as the mean ± SEM; n=6/group; ns, no significant difference; ^*^P < 0.05.

Neuronal cell death was detected by terminal deoxynucleotidyl transferase-mediated dUTP nick-end labeling (TUNEL) staining [31]. In the present study, TUNEL and NeuN coimmunofluorescence staining was conducted in the cortex and hippocampal CA1 regions to evaluate neuronal cell death after MCAO surgery (Figure 4A). Our results indicated that CHIP overexpression significantly reduced the number of TUNEL-positive (TUNEL^+^) cells in the cortex and hippocampal CA1 regions after MCAO surgery (Figure 4B), which indicated that CHIP overexpression could reduce the neuronal cell death caused by CIR.

### CHIP overexpression attenuated necroptosis induced by MCAO

**Figure 5:**
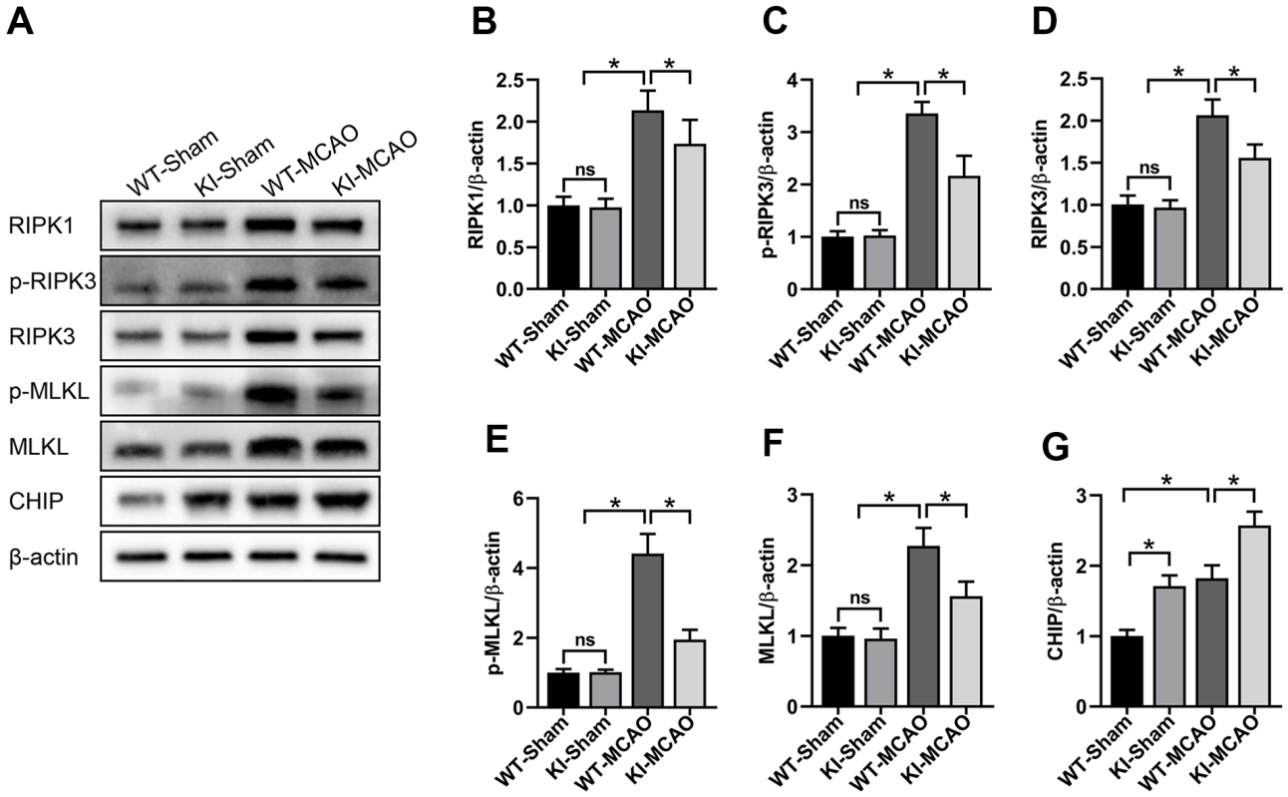
**CHIP overexpression attenuated necroptosis induced by MCAO.** (**A**) Western blot of RIPK1, p-RIPK3, RIPK3, p-MLKL, MLKL, and CHIP in the peri-infarct tissues after MCAO. (**B**–**F**) Quantitative analysis of RIPK1, p-RIPK3, RIPK3, p-MLKL, and MLKL in the peri-infarct tissues after MCAO. (**G**) Quantitative analysis of CHIP. Data are presented as the mean ± SEM; n=6/group; ns, no significant difference; ^*^P < 0.05.

Necroptosis is regulated by the RIPK1/RIPK3/MLKL axis. The formation of phosphorylated RIPK3 (p-RIPK3) and phosphorylated MLKL (p-MLKL) are crucial stages in necroptosis progression and are considered hallmarks of necroptosis [32]. Thus, we checked RIPK1, RIPK3, and MLKL expression and p-RIPK3 and p-MLKL levels by western blotting to examine necroptosis after MCAO surgery (Figure 5A). Our results showed that the RIPK1, RIPK3, and MLKL levels were significantly increased in the peri-infarct tissues after MCAO surgery compared with sham surgery (Figure 5B, 5D, 5F). Concurrently, the expression of RIPK1, RIPK3, and MLKL in the CHIP KI mouse group was markedly reduced compared with that in the WT mouse group after MCAO surgery (Figure 5B, 5D, 5F). Western blotting results of p-RIPK3 and p-MLKL showed a similar trend (Figure 5C, 5E). CHIP levels in different groups are shown in Figure 5G. These results indicated that CHIP overexpression attenuates necroptosis induced by MCAO surgery.

### CHIP overexpression alleviated inflammation after MCAO

**Figure 6:**
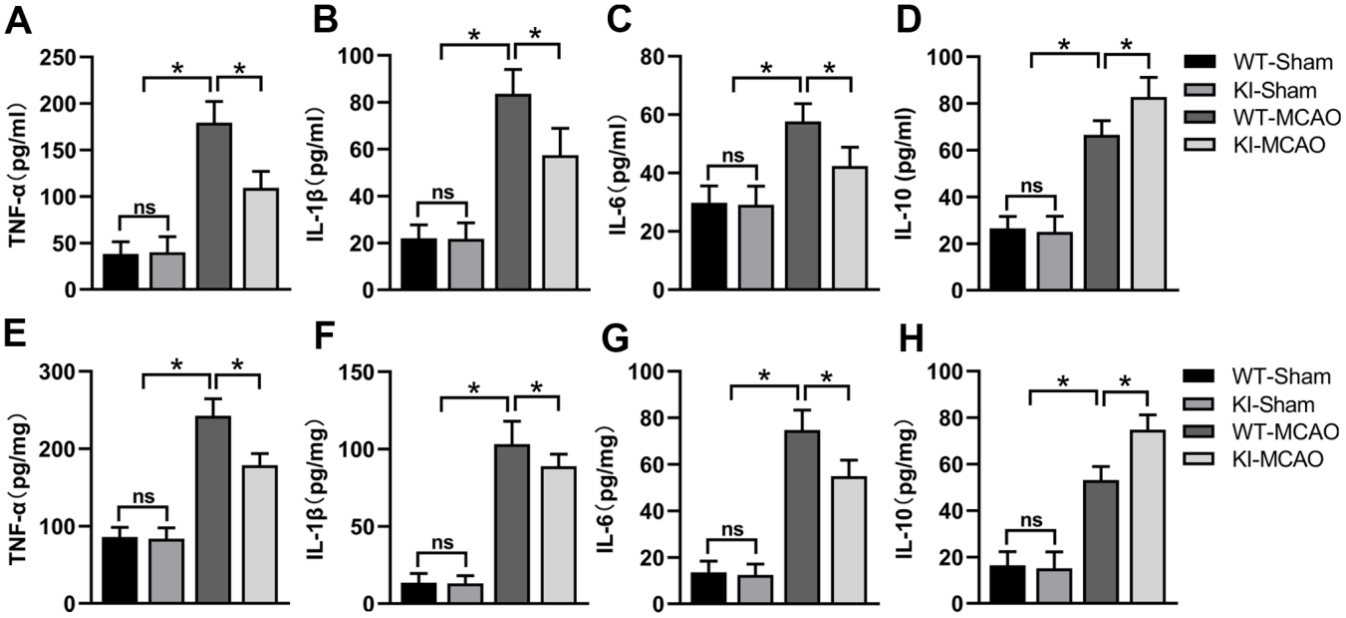
**CHIP overexpression alleviated the inflammatory response after MCAO.** (**A**–**D**) Proinflammatory mediators (TNF-α, IL-1β, IL-6) and anti-inflammatory mediator (IL-10) levels in the serum after MCAO. (**E**–**H**) Pro-inflammatory mediators (TNF-α, IL-1β, IL-6) and anti-inflammatory mediator (IL-10) in peri-infarct tissues after MCAO. Data are presented as the mean ± SEM; n=6/group; ns, no significant difference; ^*^P < 0.05.

Inflammation contributes to brain injury following CIR. After ischemia-reperfusion, a variety of cells in brain tissue are active and release inflammatory mediators, triggering local and systemic inflammatory responses. To evaluate the effect of CHIP overexpression on inflammation after MCAO, we tested the levels of proinflammatory cytokines (TNF-α, IL-1β, and IL-6) and anti-inflammatory cytokine (interleukin-10, IL-10) in the serum and brain tissues by enzyme-linked immunosorbent assay (ELISA). Our results showed that the levels of TNF-α, IL-1β, and IL-6 were significantly increased in the serum and peri-infarct tissues after MCAO surgery (Figure 6A–6C, 6E–6G). Conversely, CHIP overexpression reduced TNF-α, IL-1β, and IL-6 levels after MCAO surgery (Figure 6A–6C, 6E–6G). Furthermore, the level of IL-10 was increased in the serum and peri-infarct tissues after MCAO, and CHIP KI mice had a higher level of IL-10 than WT mice after MCAO surgery (Figure 6D, 6H). These results suggested that CHIP overexpression exerts an anti-inflammatory effect in the MCAO mouse model.

## DISCUSSION

In the present study, we constructed CHIP KI mice overexpressing CHIP in neural tissue and clarified the neuroprotective effects of CHIP on CIR injury using MCAO models with WT mice and CHIP KI mice. Our results indicated that CHIP expression was upregulated in the peri-infarct tissue after MCAO surgery. CHIP overexpression significantly ameliorated brain injury induced by MCAO surgery, such as improved neurological deficit scores, reduced cerebral infarct volume, and alleviated brain edema and neuronal impairment. Moreover, CHIP overexpression attenuated necroptosis and inflammation after MCAO surgery. These results indicated that the neuroprotective effects of CHIP might be due to its inhibitory effects on necroptosis and inflammation after MCAO surgery.

Previous studies have shown that CHIP overexpression exerts neuroprotective effects on some chronic neurodegenerative diseases by increasing the degradation of abnormal proteins [33, 34]. For example, CHIP overexpression mitigates memory deficits in an Alzheimer’s disease mouse model by increasing the degradation of β-amyloid protein and tau protein [35]. In addition, CHIP overexpression also has protective effects against cardiac ischemic injury [36] and some malignancies [37]. These findings indicate that upregulation of CHIP may have protective effects against multiple diseases. In the present study, we examined the role of CHIP in CIR injury, an acute neurodegenerative disease. Our results suggested that CHIP overexpression significantly ameliorated CIR injury, similar to the previous finding that CHIP overexpression exerts neuroprotective actions on acute brain injury [38]. Moreover, our previous study also indicated that increasing CHIP by anisomycin, a c-Jun N-terminal kinase agonist, can improve cell viability in an OGD cell model [25]. However, Stankowski et al. reported that chronic CHIP overexpression can be harmful to cell survival, while downregulation of CHIP expression enhanced neuronal tolerance to oxidative stress [39]. In addition, some studies have also indicated that CHIP can promote or suppress tumor progression in different tumors via different molecular mechanisms [40, 41]. Overall, these findings suggest the sophisticated functions of CHIP and different disease models can respond differently to CHIP levels.

Necroptosis, a form of programmed cell death, is induced by a series of protein kinases and is mainly regulated by RIPK1, RIPK3, and MLKL kinases. Multiple pathological conditions involve necroptosis, such as ischemia-reperfusion injury and inflammation. Studies have indicated that RIPK1 or RIPK3 inhibitors, such as necrostatin-1 (RIPK1 inhibitor) [12] and GSK’872 (RIPK3 inhibitor) [42], can protect against brain injury by blocking necroptosis after MCAO. RIPK1, RIPK3, and MLKL play a pivotal role in inducing necroptosis, and RIPK1, RIPK3, and MLKL upregulation could be common in many neurological diseases [43], including CIR injury [44]. The presence of p-RIPK3 and p-MLKL is considered a hallmark of necroptosis. Previous study has shown that CHIP attenuates necroptosis through the ubiquitylation of RIPK1 and RIPK3. Increasing CHIP levels can improve cell viability in an OGD cell model by attenuating necroptosis. In the present study, to verify the effect of CHIP on necroptosis after CIR, we constructed MCAO mouse models and detected RIPK1, RIPK3, MLKL, p-RIPK3, and p-MLKL levels. Our results indicated that RIPK1, RIPK3, and MLKL expression was significantly increased after MCAO surgery compared with sham surgery, and CHIP overexpression reduced RIPK1, RIPK3, and MLKL levels after MCAO surgery. In addition, p-RIPK3 and p-MLKL levels showed a similar trend. These results indicated that CHIP overexpression attenuates necroptosis following CIR.

Inflammation is an essential factor leading to the aggravation of brain injury caused by CIR, and reducing inflammation can mitigate brain injury after CIR. Studies have shown that inflammatory responses of damaged brain tissues involve different mechanisms, such as the activation of immune cells and necroptosis after CIR. In contrast to apoptosis, necrotic cells can release DAMPs, promoting inflammation during necroptosis. Thus, necroptosis is also considered a proinflammatory cell death mode, and blocking necroptosis can decrease inflammation [45]. Furthermore, study has shown that proinflammatory mediators, such as TNF-α, IL-1β, and IL-6, and anti-inflammatory mediators, such as IL-10, are involved in the inflammatory responses induced by CIR [46]. In the present study, we tested the levels of proinflammatory (TNF-α, IL-1β, and IL-6) and anti-inflammatory (IL-10) cytokines in the serum and peri-infarct tissues to determine whether CHIP exerts anti-inflammatory effects in MCAO mouse models. Our results showed that CHIP overexpression markedly decreased proinflammatory cytokines and increased anti-inflammatory cytokine levels, suggesting that CHIP overexpression has an anti-inflammatory effect after CIR.

Moreover, it is necessary to note that increasing CHIP levels by gene KI is difficult for clinical practice. Therefore, we need to find other ways to increase CHIP levels effectively and use them for clinical application. To date, several small-molecule agonists have been reported to upregulate CHIP expression in cell models, such as anisomycin [25], peptidoglycan [47], and 2-(4-hydroxy-3-methoxyphenyl)-benzothiazole [48]. However, it still needs to be explored whether these small-molecule agonists can upregulate CHIP expression in the brain and mitigate the brain injury induced by CIR. The adeno-associated viral vector is an effective CNS gene delivery method that can cross the blood-brain barrier [49]. Our previous study showed that adeno-associated viruses carrying the CHIP gene can upregulate CHIP expression in the mouse brain and exert protective effects in a Parkinson’s disease mouse model [50], which showed that the adeno-associated viral platform may be an efficient way to upregulate CHIP expression in the CNS. Overall, developing a safe and effective method that can induce CHIP overexpression in the CNS for CIR injury treatment may be the focus of future research.

Collectively, our study suggests that CHIP was induced in mouse brain tissue following experimental CIR, and CHIP overexpression ameliorates brain injury by attenuating necroptosis and inflammation following CIR. Taken together, CHIP may be a promising therapeutic agent for CIR injury, and its clinical application requires further research.

## MATERIALS AND METHODS

### Animals

Adult male mice (aged 10–12 weeks, weighing 25–30 g; Henan Experimental Animal Center, Henan, China), including WT C57BL/6 mice and CHIP KI mice with a C57BL/6 background, were used in this study. CHIP KI mice were constructed as described in our previous study [50]. Briefly, CRISPR/Cas9 technology was used to construct CHIP-with-floxed-STOP-codon mice, which were crossed with Nestin-cre mice to obtain NES-CHIP mice (we named CHIP KI mice here). CHIP-KI mice can selectively increase CHIP expression in neural tissues. All mice were maintained in an environment with controlled light (12 h light/dark), humidity 55%–60%, temperature 22 ± 1° C, and group-housed at 4–5 per cage. All experiments were reviewed and approved by the Institutional Animal Care and Use Committee of Zhengzhou University, China. All experimental procedures were performed according to the guidelines of the Ethics Committee of The First Affiliated Hospital of Zhengzhou University for the care and use of experimental animals.

### MCAO model

The MCAO mouse models were generated using the intraluminal filament technique [51]. Briefly, the mice were anesthetized with sodium pentobarbital and then transferred to an operating table, and their body temperatures were maintained at 37 ± 0.5° C. Next, the iodine complex was used to disinfect the incision site of the neck skin, and a midline incision at the neck was made to separate the right common carotid artery, right external carotid artery, and right internal carotid artery. A silicone-coated nylon microfilament (#L2000, Guangzhou Jialing Biotechnology Company, Guangzhou, China) was inserted into the right internal carotid artery through the right external carotid artery incision to occlude the right middle cerebral artery. The filament was withdrawn for reperfusion after it remained in position for 1 h. The mice were subjected to the same procedures in the sham surgery groups, except the right middle carotid artery was not occluded.

### Experimental design

This study consisted of four parts. The first part studied the expression level of CHIP after CIR. WT mice were subjected to sham or MCAO surgery and euthanized at separate time points after reperfusion, and their brain tissues were used for western blotting, RT-PCR, and histological analyses. The second part compared the expression levels of CHIP between WT mice and CHIP KI mice. The mouse brains were harvested for western blotting, RT-PCR, and histological analysis. The animals were separated into four groups and subjected to sham or MCAO surgery in the third part and the fourth part: WT-Sham (WT mice subjected to sham surgery), KI-Sham (CHIP KI mice subjected to sham surgery), WT-MCAO (WT mice subjected to MCAO surgery), and KI-MCAO (CHIP KI mice subjected to MCAO surgery). The third part studied whether CHIP overexpression has neuroprotective roles in MCAO mouse models. Each group of animals was euthanized at 24 h after reperfusion. Their brain tissues were used for TTC staining, HE staining, Nissl staining, brain edema experiments, and TUNEL staining analyses. The fourth part examined necroptosis and inflammation after MCAO. In all experiments, the examiners were blinded to the mouse genotypes of all groups.

### Neurological scores

The neurological deficits of mice were assessed according to the Longa test [52]. Briefly, the scoring criteria consisted of 0, 1, 2, 3, and 4 points, with no neurological deficits = 0 and maximal deficits = 4. Consequently, higher scores indicate worse injury.

### Infarct volume

The cerebral infarct volume was measured using TTC staining. Briefly, the brain slices were incubated with 2% TTC solution (#G3005, Solarbio, Beijing, China) for 15 min at 37° C without lighting. Next, the brain slices were fixed in paraformaldehyde (#G1101, Servicebio, Wuhan, China) overnight and then photographed. TTC staining results were quantified using ImageJ software (National Institutes of Health, USA).

### Brain edema

Brain edema was assessed by brain water content, calculated by the dry-wet weight method [53]. Briefly, at 24 h after reperfusion, mice were deeply anesthetized, and their brains were harvested quickly, sectioned along the midsagittal plane, and separated into the ischemic hemisphere (ipsilateral hemisphere) and nonischemic hemisphere (contralateral hemisphere). Each hemisphere was weighed immediately and then dried to obtain the wet weight and dry weight. The brain water content was calculated as [(wet weight − dry weight)/wet weight] × 100%.

### Tissue preparation

The animals were deeply anesthetized following sham or MCAO surgery and subjected to different processing methods. For histological analysis, after continuous cardiac perfusion with phosphate buffer saline (PBS) and paraformaldehyde (#G1101, Servicebio), the mouse brains were extracted and fixed in paraformaldehyde overnight, dehydrated with 30% sucrose solution, and finally sectioned at 20 μm thickness. For western blot and RT-PCR analyses, the brain tissues were removed quickly and stored at −80° C after sham or MCAO surgery.

### HE and Nissl staining

Histological features of the infarct areas were assessed using HE staining (#BA-4224, Baso, Zhuhai, China) and Nissl staining (#G1430, Solarbio) based on the manufacturer’s instructions. For HE staining, slices were washed in PBS to dissolve the tissue freezing medium and then submerged in hematoxylin for 3 min, washed with running water for 10 s and submerged in eosin solution for 45 s. After that, slices were dehydrated in a gradient ethanol solution, cleared in xylene, and covered with coverslips. For Nissl staining, slices were successively incubated in cresyl violet solution for 45 min at 56° C and differentiation solution for 1 min at room temperature (RT). Finally, slices were dehydrated in a gradient ethanol solution, cleared in xylene, and covered with coverslips. The images were recorded using an electron microscope (Leica DMi8, Germany).

### Immunofluorescence and TUNEL staining

Slices were washed in PBS for 15 min (total 3 times, 5 min each time), and then antigen retrieval was performed using citrate antigen retrieval solution (#P0081, Beyotime, Shanghai, China) at 100° C. After that, slices were permeabilized with 0.3% Triton X-100 for 30 min and incubated with 5% normal goat serum for 60 min at RT. Slices were then incubated at 4° C overnight (approximately 12 h) with primary antibodies, including CHIP (1:200, #ab134064, Abcam) and NeuN (1:1000, #ab104224, Abcam). After that, the slices were incubated with the corresponding fluorescent antibodies, Alexa Fluor 555-labeled Donkey Anti-Rabbit IgG (1:500, #A0453, Beyotime) and Alexa Fluor 488-labeled Goat Anti-Mouse IgG (1:500, #A0428, Beyotime) for 2 h at RT. Nuclei were stained with 4′,6-diamidino-2-phenylindole (DAPI).

TUNEL (#A112-03, Vazyme Biotech, Nanjing, China) staining was used to detect neuronal cell death with DNA breakage. Coimmunostaining of TUNEL, NeuN, and DAPI was performed as previously described [54]. ImageJ software (NIH, USA) was used to analyze fluorescence images.

### Western blot analysis

Brain tissues were homogenized to extract total protein. Equal amounts of protein were separated on 10% SDS-PAGE gels (#PG112, Epizyme, Shanghai, China) and then transferred to PVDF membranes (#IPFL00005, Millipore, MA, USA). After blocking with 5% bovine serum albumin for 2 h at RT, the membranes were incubated with primary antibodies against CHIP (1:10000, #ab134064, Abcam), RIPK1 (1:1000, #SAB3500420, Sigma-Aldrich), RIPK3 (1:1000, #ab62344, Abcam), MLKL (1:2000, #orb32399, Biorbyt), p-RIPK3 (1:1000, #AF7443, Affinity), p-MLKL (1:1000, #AF7420, Affinity), and β-actin (1:5000, #380624, ZEN BIO) at 4° C overnight. After that, the membranes were incubated with HRP-conjugated Goat Anti-Rabbit IgG (H+L) (1:5000, #SA00001-2, Proteintech) for 2 h at RT and then treated with enhanced chemiluminescence reagents for visualization using Image Lab software (BIO-RAD, USA).

### RT-PCR analysis

Total RNA was isolated from brain tissue using an RNA isolation kit (#R401-01, Vazyme Biotech). A total of 1 μg RNA from each sample was reverse transcribed into cDNA using a cDNA synthesis kit (#R223-01, Vazyme Biotech). RT-PCR was performed using a ChamQ Universal SYBR qPCR Master Mix Kit (#Q711-02, Vazyme Biotech) on QuantStudio 5. The primer sequences were as follows: CHIP forward: 5′-CGGCAGCCCTGATAAGAGC-3′; CHIP reverse: 5′- CACAAGTGGGTTCCGAGTGAT-3′. β-actin forward: 5′- GGCTGTATTCCCCTCCATCG-3′; β-actin reverse: 5′- CCAGTTGGTAACAATGCCATGT-3′. Relative mRNA expression was quantified using the 2^-ΔΔCt^ method, with β-actin serving as the internal control.

### ELISA

The brain tissues were homogenized in PBS to obtain the tissue fluid. Blood samples were placed in sterile tubes for 1 h at RT and then centrifuged at 2000×g for 10 min at 4° C to obtain serum. ELISA kits were used to detect the concentrations of TNF-α (#E-EL-M0049c, Elabscience, Wuhan, China), IL-1β (#E-EL-M0037c, Elabscience), IL-6 (#E-EL-M0044c, Elabscience), and IL-10 (#E-EL-M0046c, Elabscience) according to the manufacturer’s instructions.

### Statistical analyses

SPSS software (version 26.0; IBM Corp. USA) was used for data analysis. All data are expressed as the mean ± SEM. Two groups were compared using Student’s t test, whereas analyses of multiple groups were performed using one-way analysis of variance followed by Tukey’s post hoc test. Statistical significance was defined as a p value of < 0.05.
